# *Lactobacillus acidophilus* (strain Scav) postbiotic metabolites reduce infection and modulate inflammation in an *in vivo* model of *Pseudomonas aeruginosa* wound infection

**DOI:** 10.1093/jambio/lxaf061

**Published:** 2025-03-11

**Authors:** Rachael M Wilson, Jean M Walker, Joris Beld, Kingsley Yin

**Affiliations:** Department of Cell Biology and Neuroscience, Rowan-Virtua School of Translational Biomedical Engineering and Sciences, Virtua Health College of Medicine and Life Sciences, Stratford, NJ 08084, USA; Department of Cell Biology and Neuroscience, Rowan-Virtua School of Translational Biomedical Engineering and Sciences, Virtua Health College of Medicine and Life Sciences, Stratford, NJ 08084, USA; Department of Microbiology and Immunology, College of Medicine, Drexel University, Philadelphia, PA 19104, USA; Department of Cell Biology and Neuroscience, Rowan-Virtua School of Translational Biomedical Engineering and Sciences, Virtua Health College of Medicine and Life Sciences, Stratford, NJ 08084, USA

**Keywords:** *Lactobacillus acidophilus*, postbiotic, *Pseudomonas aeruginosa*, antibiofilm, metabolite characterization, wound infection

## Abstract

**Aims:**

This study assessed the antibacterial, antibiofilm, and immunomodulatory activity of *Lactobacillus acidophilus* (strain Scav) postbiotic (LaP) in a mouse model of *Pseudomonas aeruginosa* wound infection and evaluated the bioactive components of the LaP.

**Methods and results:**

LaP was tested for *Pseudomonas aeruginosa* clearance and immunomodulatory activity during wound infection. We show that LaP applied 1 h after infection reduced tissue bacterial burden within 24 h, and this reduction persisted for 5 days. Ciprofloxacin given once at the exact same time did not reduce bacteria load as compared to vehicle controls. LaP reduced plasma IL-6 and MCP-1 levels after 5 days. Wound tissue IL-6 and MCP-1 levels were increased in infected vehicle mice at 5 days, but tissues from LaP-treated mice were similar to sham controls. LaP increased tissue IL-10 (antiinflammatory cytokine) levels. Ciprofloxacin decreased plasma and tissue IL-6 compared to vehicle controls but did not affect MCP-1 or IL-10 levels. To elucidate antibacterial and antibiofilm metabolite(s) in LaP, fractionation followed by *Ps. aeruginosa* antagonistic activity assays were performed. This was followed by liquid chromatography coupled to mass spectrometry (LCMS) analysis. Our analyses identified a low molecular weight, polar molecule, which had both antibacterial and antibiofilm activity.

**Conclusions:**

*Lactobacillus acidophilus* secretes an antibacterial and antibiofilm metabolite that reduced pathogen burden and resolved systemic inflammation in a *Pseudomonas aeruginosa* wound infection model.

Impact Statement These findings suggest that *Lactobacillus acidophilus* secretes an antibacterial and antibiofilm molecule that is a promising candidate for treatment of severe and virulent infections.

## Introduction

Acute and chronic wound infections pose a healthcare challenge with increasing morbidity and mortality rates (Siddiqui and Bernstein 2010, Pondei et al. 2013, Falcone et al. 2021). Wound healing is a coordinated process that involves hemostasis, inflammation, proliferation, and remodeling (Rodrigues et al. 2019, Raziyeva et al. 2021). During normal wound healing, immune cells are recruited to the injury to clear tissue debris and prevent infection. Specifically, proinflammatory mediators such as immune cell chemoattractants, cytokines, reactive oxygen species, and prostaglandins are produced to promote wound healing. However, sustained inflammation may lead to lymphocyte death, reduced antigen presentation, and the inability of monocytes/macrophages to secrete inflammatory cytokines when stimulated (Hesketh et al. 2017, Larouche et al. 2018, Kim and Nair 2019, Rodrigues et al. 2019, Joshi et al. 2020, Wilkinson and Hardman 2020, Raziyeva et al. 2021). In this state of immunosuppression, the infection may persist, and the host is susceptible to infection. If the wound becomes infected, wound healing is disrupted (Wilkinson and Hardman 2020). *Pseudomonas aeruginosa* a Gram-negative opportunistic pathogen responsible for hospital-acquired infections, such as in respiratory and urinary tracts, readily forms biofilm and is associated with high mortality rates (Mulcahy et al. 2008, Zhang et al. 2020). Importantly, the bacterium has been isolated from wound infections (Gjodsbol et al. 2006, Kirketerp-Moller et al. 2008, Pondei et al. 2013, Lenzmeier et al. 2019, Puca et al. 2021).

*Pseudomonas aeruginosa* has several virulence factors that promote its survival and antibiotic resistance, including endotoxin and pyocyanin release as well as biofilm formation (Liao et al. 2022). Bacterial cells in the biofilm are encapsulated within a matrix of extracellular polymeric substances (EPS), aiding antibiotic resistance (Zhou et al. 2021, Fernández-Billón et al. 2023). In particular, the EPS matrix consists of lipids, proteins, nucleic acids, and polysaccharides that support the bacteria to have altered metabolic functions, possibly leading to persister cells (Peters et al. 2022). As a result, bacteria within the biofilm commonly resist antibiotic treatment through multiple mechanisms. First, antibiotic tolerance may arise because bacteria within a biofilm undergo altered cell division, a target of many antibiotics (Ronneau et al. 2021, Armes et al. 2022). Another possibility is that biofilm bacteria have increased genetic transfer, where plasmids carrying resistance genes can be transferred to other bacterial cells (Sharma et al. 2017). Standard treatment modalities for biofilm aim to kill the bacteria and/or inhibit biofilm formation (Verderosa et al. 2019). Conventional antibiotics remain the most common therapy, although alternative drug delivery systems such as nanoparticles and photodynamic therapy have gained interest (Ciarolla et al. 2022, Mishra et al. 2023). With respect to wound infections, topical dressings such as silver sulfadiazine and nanocrystalline silver remain common therapies (Chappell and Nair 2020). Still, biofilm recalcitrance persists and there is a great need for treatments that can target both planktonic cells within the biofilm and destroy the EPS. Alarmingly, antibiotics may lower bacterial activity and lead to a persister cell phenotype within the biofilm, which is difficult to eradicate with antibiotics (Clinton and Carter 2015, Ronneau et al. 2021, Peters et al. 2022). As such, there is a clear need for antibiotic alternatives that can penetrate biofilm to clear infection and promote wound healing.

Probiotics are known to have antiinflammatory, barrier function, and antimicrobial abilities (Latif et al. 2023). Specifically, there are many reports documenting that probiotics had beneficial actions in rodent models of colitis, anaphylaxis, burn wound infection, drug-resistant *Ps. aeruginosa* infection, and sepsis (Chen et al. 2009, Khailova et al. 2013, Li et al. 2013, Machairas et al. 2015, Argenta et al. 2016, Din et al. 2020, Wang et al. 2020). In these studies, the beneficial effects were observed after prophylactic probiotic administration and, therefore, did not examine the therapeutic value of probiotics given after infection. Moreover, many of these studies focused on using whole cell suspensions of probiotic bacteria, and typically suggest their role in preventing disease. In particular, Mekky et al. (2022) reported that *Lactobacillus plantarum* inhibited the formation of multidrug-resistant (MDR) *Escherichia coli* U12 when pretreated or cocultured. Similarly, *Lact. casei* and *Lact. acidophilus* prevented *Ps. aeruginosa* biofilm formation but surprisingly did not have direct antibacterial activity against *Ps. aeruginosa* planktonic cells (Diaz et al. 2020). On the other hand, treatment of macrophages with *Lactobacillus* spp. activated cytokine production (Rocha-Ramirez et al. 2017), but cell-free *Lactobacillus* supernatant was antiinflammatory in LPS-activated macrophages (De Marco et al. 2018).

We have recently reported that *Lact. acidophilus* postbiotic (LaP) had dose-dependent effects on monocyte and macrophage NF–κB activity (Wilson et al. 2021). Interestingly, in quiescent THP-1 monocytes, high concentration LaP stimulated NF–κB activity, while low concentration LaP downregulated NF–κB activation in LPS-pretreated macrophages. Together, these findings suggest that our preparation of LaP had an immunomodulatory action on mononuclear cells. We also showed that LaP was bactericidal against *Ps. aeruginosa* and removed established preformed *Ps. aeruginosa* biofilm (Wilson et al. 2021). The bioactive components of our LaP were not determined and are therefore investigated in the present study.

There has been a surge in studying the mechanisms of action of probiotics, and several lines of evidence indicate that the antibacterial properties are due to the secretion of metabolites, including organic acids, short-chain fatty acids, and antimicrobial peptides (AMPs), including bacteriocins (Tan et al. 2014,Peng and Biswas 2017). Nevertheless, most studies using bacteriocins have been conducted *in vitro*, and the safety, efficacy, and bioavailability *in vivo* are not entirely understood (Benítez-Chao et al. 2021). For example, oral administration and topical application may not be ideal since the digestive tract and skin present harsh environments (i.e. low pH and enzymatic degradation). Similarly, it has been reported that probiotic bacteria produce conjugated linoleic acid (CLA) and conjugated linoleic acid isomers from free linoleic acid (LA) in the gut that have both antibacterial and antiinflammatory properties (Bassaganya-Riera et al. 2012).

In the present study, we hypothesized that direct topical application of LaP can reduce wound bacteria load and modulate systemic inflammatory response when given a full hour after infection in a *Ps. aeruginosa* model of wound infection. In addition, we aimed to elucidate the identity of the antibacterial and antibiofilm metabolite. Specifically, biochemical and bioactivity assays were used in tandem to fractionate the antibiofilm molecule. These are the first studies designed to simultaneously investigate both the antimicrobial and immunomodulatory properties of LaP given after wound infection.

## Materials and methods

### *Lactobacillus acidophilus* cultivation and postbiotic collection

LaP was collected from *Lact. acidophilus (*ATCC 4356) as previously described with minor deviation (Wilson et al. 2021). Briefly, bacteria were cultivated in De Man, Rogosa, and Sharpe (MRS) broth (Research Products International, Mt. Prospect, IL, USA) as recommended by ATCC, for 48 h at 37°C, 5% CO_2_, and subcultured in 10 ml M63 minimal medium supplemented with 0.2% glucose, 1 mM MgSO_4_, and 0.5% casamino acids (Fisher BioReagents, Pittsburgh, PA, USA) for 6 h. Culture supernatants were passed through 0.2 µm filters to collect LaP. To determine the number of colony forming units (CFUs) within cultures prior to LaP collection, serial dilutions were made in sterile saline (Molecular Biologicals International, Irvine, CA, USA) and spread onto MRS agar, incubated at 37°C for 48 h, and colonies numerated. In these studies, LaP was collected from 1 × 10^6^ CFU ml^−1^ (L LaP) and 1 × 10^8^ CFU ml^−1^ (LaP). For solid-phase extraction (SPE) and semipreparative High Performance Liquid Chromatography (HPLC), *Lact. acidophilus* LaP was scaled-up to 1 l.

### *Pseudomonas aeruginosa* preparation

*Pseudomonas aeruginosa* was prepared as previously described (Wilson et al. 2021). In brief, *Ps. aeruginosa* (ATCC 27853) was cultivated on tryptic soy agar (TSA; Ward’s Scientific, Rochester, NY, USA) overnight at 37°C. Single colonies were inoculated in Luria–Bertani (LB) broth (Gibco, Gaithersburg, MD, USA) and incubated for 5 h with shaking. Cultures were pelleted, washed with M63 media, and diluted to OD_600_ between 0.04 and 0.06 (1 × 10^6^–5 × 10^6^ ml^−1^) using a BioTek Synergy H1 plate reader (Biotek, Winooski, VT, USA). Here, M63 media was prepared to mimic the acidic condition of human skin by adjusting to pH 5.5 (Bullock et al. 2020, Wilson et al. 2021).

### Planktonic growth curves and CFU counts

For antibacterial measurement against planktonic *Ps. aeruginosa*, 100 µl of LaP prepared in M63 was pipetted into the wells of 96-well plates inoculated with 100 µl *Ps. aeruginosa* in triplicate as previously described (Wilson et al. 2021). Planktonic growth was measured every 10 min for 18 h in a plate reader (BioTek Synergy H1) set to 37°C with orbital shaking. Following growth curves, viable *Ps. aeruginosa* cell numbers were enumerated as previously reported (Wilson et al. 2021). In brief, cell cultures were recovered from the wells, serially diluted, spread onto TSA, incubated overnight at 37°C, and colonies counted for *n* = 4 independent experiments.

### Antibiofilm activity assay

The biofilm removal assay (Wilson et al. 2021) was adopted for testing the antibiofilm activity of crude LaP and LaP fractions (in duplicate) against 20 h *Ps. aeruginosa* established biofilm. *Pseudomonas aeruginosa* was propagated on TSA and incubated at 37°C. Single colonies were inoculated in LB broth for 5 h with shaking. The cultures were washed and diluted to 1 × 10^8^ CFU ml^−1^ in M63 minimal medium. 200 µl of *Ps. aeruginosa* culture was pipetted into a round-bottom 96-well plate and incubated at 37°C for 20 h. Planktonic cells were washed away using phosphate-buffered saline (PBS; GrowCells, Irvine, CA, USA) and 200 µl of indicated treatments in quintuplicate were added for 6 h. Biofilm biomass was quantified for three to four independent experiments by using crystal violet staining and OD_600_ was measured on a BioTek H1 Synergy plate reader. Here, antibiofilm activity was used to determine the efficacy of crude LaP on various fractions for bioactivity to elucidate a bioactive metabolite.

### Wound infection surgery

To examine the antibacterial effect and wound healing properties of LaP in *Ps. aeruginosa* wound infection, male and female C57BL/6 mice (20–30 g; Envigo, Somerset, NJ, USA) were used. As previously described (Wilson 2023, Fig. 2A), animals were anesthetized with isoflurane (+ 3% O_2_) and received subcutaneous injections of Buprenorphine XR (Ethiqa; 3.25 mg kg^−1^) (Fidelis Animal Health Inc., North Brunswick, NJ USA). Hair was removed, and a full thickness wound was created on the back using a disposable 6 mm biopsy punch (Integra Miltex, Mansfiled, MA, USA). 30 µl of *Ps. aeruginosa* in saline (1 × 10^7^ CFU; ATCC #27853) was applied directly on the wound and covered with Tegaderm transparent dressing (3 M Health Care, St. Paul, MN, USA). 1 h following surgery, mice were anesthetized and received 30 µl of LaP (derived from 1 × 10^8^ CFU ml^−1^) treatment or M63 vehicle under the Tegaderm, directly on the wound. In some mice which were scheduled as 5 day infection animals, ciprofloxacin (250 mg kg^−1^) was injected once subcutaneously 1 h after wound infection surgery. We chose this regimen so that it was comparable to the single dose administration of the LaP. In sham controls, wounds were created but not inoculated with *Ps. aeruginosa*. After 24 h or 5 days, mice were anesthetized with ketamine/xylazine (100/10 mg kg^−1^, i.p.). Blood was drawn via intracardiac puncture into tubes containing 50 mmol l^−1^ ethylenediaminetetraaceticacid (EDTA). Plasma was collected after centrifugation at 1000 × *g* for 10 min. The full thickness wounds were excised with underlying muscle and homogenized in PBS. Wound tissue samples were used for viable bacterial counts and enzyme-linked immunosorbent assay (ELISA) analysis. For 24 h experiments, we used 5 sham, 14 vehicle, and 14 LaP. For 5 day studies, we used 5 sham, 13 veh, 11 LaP, and 5 ciprofloxacin mice. For each of the assays, the numbers of mice in each group are stated in the figure legends. All procedures were performed with protocols approved by the Institutional Animal Care and Use Committee of Rowan-Virtua School of Osteopathic Medicine.

### Bacterial load quantification

At 24 h and 5 days, full thickness wounds were excised and homogenized as described above. To enumerate wound bacterial colonization and clearance, tissues were diluted in PBS, plated on TSA, incubated overnight at 37°C, and colonies counted. Tissue sample protein concentration was quantified using Coomassie Plus Protein Assay Reagent (Thermo Scientific, Rockford, IL, USA). Bacterial CFUs in wound tissue were standardized to CFU mg^−1^ protein (Buyk et al. 2015). Similarly, blood was saline diluted, spread on TSA, incubated overnight, and colonies counted.

### Inflammatory mediator analysis

MCP-1, IL-6, and IL-10 levels in frozen homogenized tissue samples as well as MCP-1 and IL-6 in plasma samples were quantified using ELISA (Thermo Fisher Scientific, Waltham, MA, USA). Protein levels in wound tissue was determined as described above, and tissue MCP-1, IL-6, and IL-10 concentrations were normalized to pg mg^−1^ total protein.

### Monocyte adherence assay

THP-1 (ATCC TIB-202™) monocytes were maintained as described in detail in Wilson et al. 2021. Monocyte adherence assay was conducted by blocking laminin-coated 24-well plates (Corning, Manassas, VA, USA) with 0.1% bovine serum albumin (BioVision, Milpitas, CA, USA) for 1 h at 37°C, 5% CO_2_ and washed with PBS. THP-1 monocytes were seeded at 3 × 10^5^ cells per well in serum- and pen/strep-free RPMI (reduced RPMI) in triplicate. To elucidate the effect of LaP on monocyte adherence, monocytes were exposed to 50 µl *Lact. acidophilus* LaP (derived from 1 × 10^8^ CFU ml^−1^), M63 vehicle, or 100 ng ml^−1^ phorbol 12-myristate 13-acetate (PMA; Sigma-Aldrich, St. Louis, MO, USA). Following 1 h incubation, nonadherent cells were removed and plates were washed with saline. Monocytes were replenished with reduced RPMI and stained with 10 µl of CellBrite Blue Cytoplasmic stain (Biotium, Fremont, CA, USA) for 20 min. The plates were washed with saline and RPMI was replenished. Adherent cells were imaged using a Keyence BZ-X7010 and the number of cells mm^−2^ was quantified using ImageJ software for *n* = 3 independent experiments.

### Crude LaP SPE

Crude LaP (derived from 1 × 10^8^ CFU ml^−1^) or M63 minimal medium control were loaded on C18 cartridge columns (Sep-Pak, Waters) conditioned with 100% acetonitrile (ACN) and equilibrated with water. LaP was eluted using a 10%–100% ACN gradient and flow through was collected. Each fraction was lyophilized using a Virtis Freezemobile 24. The lyophilized fractions were resuspended in 100 µl sterile water and diluted in M63 media. The diluted samples were examined for planktonic growth inhibition and antibiofilm activity as described above. In initial studies, the LaP was scaled-up by subculturing in 1 l M63 minimal media instead of 10 ml. To confirm that scaling-up did not hinder activity, the scaled-up crude LaP was tested for biofilm removal activity. As expected, the scaling-up of the LaP did not affect the antibiofilm activity (Fig. S2A). Therefore, the scaled-up LaP was fractionated using SPE over the 10%–100% ACN gradient. The fractions were lyophilized, resuspended in water, and subsequently diluted 1:10 and 1:100 in M63 minimal medium. Here, lyophilization was essential to remove ACN, as it has been shown to have broad-spectrum antibacterial properties (Ghanem and El-Magly 2008, Dulay et al. 2017). In addition, M63 minimal media (vehicle control) was fractionated and tested in the established biofilm assay to investigate the impact of media components on *Ps. aeruginosa* biofilm. After propagation, *Lact. acidophilus* and *Ps. aeruginosa* were resuspended and cultured in M63 minimal medium at pH 5.5 to recapitulate the acidic conditions of human skin, where *Ps. aeruginosa* commonly colonizes and forms biofilm (Percival et al. 2012, Bullock et al. 2020). However, there are several reports demonstrating the inhibitory effect of low pH on *Ps. aeruginosa* biofilm formation (Harjai et al. 2005, Hostacka and Stefkovicova 2010 et al. 2010). Therefore, we tested the effect of this fractionated acidic medium on *Ps. aeruginosa* biofilm removal. Here, no biofilm removal properties from any fraction were observed (Fig. S2B). These data validate the experimental workflow and use of M63 minimal medium in *Ps. aeruginosa* biofilm formation and subculturing and collection of *Lact. acidophilus* LaP.

### Antibiofilm metabolite purification

Following SPE, antibiofilm activity assays were performed using all different fractions as described above. Fraction 5 was determined to contain an antibiofilm molecule (Fig. 6C). To elucidate the characteristics and identity of the bioactive metabolite, SPE fraction 5 was subjected to subsequent fractionation using semipreparative HPLC. Specifically, the molecules within fraction 5 were loaded onto Phenomenex Luna Omega 5 µmol l^−1^ polar C18 250 × 12 mm column and separated using a Varian Prostar semipreparative RPHPLC system (Prostar 210 pumps, Prostar 420 autosampler, Prostar 335 diode array detector, and Prostar fraction collector). Crude LaP was purified using a 95/5 to 70/30 water/ACN containing 0.1% v/v formic acid (at 10 ml min^−1^) gradient. Following separation, the fractions were lyophilized, resuspended in sterile water, diluted in M63 minimal media, and investigated for planktonic growth inhibition and antibiofilm activity.

### Liquid chromatography coupled to mass spectrometry analysis

From semipreparative HPLC fractionation, fractions 23, 24, and 25 were selected for liquid chromatography coupled to mass spectrometry (LCMS) analysis because these fractions had the most antibiofilm activity [described in Wilson (2023) and adapted from Majer et al. (2021)]. In brief, the selected fractions were analyzed on a Waters Acquity I-Class UPLC system coupled to a Synapt G2Si HDMS mass spectrometer in positive ion mode with heated electrospray ionization (ESI) source in a Z-spray configuration. LC separation was performed on a Waters Acquity UPLC BEH C18 1.7 µm 2.1 × 100 mm column equipped with a Vanguard guard column, using an 0.6 ml min^−1^ gradient of 95/5 to 15/85 eluents A/B over the course of 4 min. Eluent A was 105 0.1% v/v formic acid in water and B was 0.1% v/v formic acid in ACN. Separately, a polar isocratic (95/5 for 4 min) was used for separation. After separation, the column was washed and reconditioned. The mass spectrometer conditions were as follows: capillary voltage 0.5 kV, sampling cone 40 V, source offset 80 V, source 120°C, cone gas 0 l h^−1^, desolvation gas 1000 l h^−1^, and nebulizer 6.5 bar. Low energy data, between 100 and 1500 Da, was collected with the analyzer in resolution mode and a 0.2 s scan time. MSe data was collected using a 20–40 V ramp trap collision energy. Masses were extracted from the Time-of Flight Mass Spectrometry Total Ion Chromatograms (TOF MS TICs) using a 0.005 Da abs width. MassLynx software (V4.1; Waters Corporation) was used to acquire and process LCMS chromatograms and spectra.

### LaP metabolite analysis

The *Lact. acidophilus* ATCC 4356 whole genome was accessed using NCBI GenBank (accession number JRUT01000000) and mined for secondary metabolite gene clusters using antiSMASH 7.0 with strictness set to “loose” and features KnownClusterBlast, Minimum Information about a Biosynthetic Gene Cluster (MIBiG) comparison (MIBiG) Comparison, ClusterBlast, Cluster Pfam, and SubClusterBlast (Majer et al. 2021). In addition, the Human Microbial Metabolome Database (MiMeDB, V 1.0) was used to screen for potential metabolites that have similar biochemical characteristics to the identified bioactive hit. MiMeDB platform was used to search for spectroscopic data by uploading the LCMS parameters and 295.1369 m/z hit.

### Statistical analyses

All data analyses and plots were performed using GraphPad Prism (San Diego, CA, USA). Biofilm removal data were subjected to one-way ANOVA and Tukey’s multiple comparisons test. For all other *in vitro* studies, data were analyzed using one-way ANOVA and Dunnett’s *post hoc* test was used to compare treatments against controls. *In vivo* bacterial load studies were analyzed using Mann–Whitney U test. For all other *in vivo* studies, differences between sham controls, vehicle controls, and LaP-treated groups were analyzed by one-way ANOVA and differences between groups were then determined using Sidak’s test. All data are presented as mean ± SEM. In all statistical analyses, *P* < .05 was regarded as significant.

## Results

### *Lactobacillus acidophilus* LaP has direct bactericidal and antibiofilm activity

**Figure 1.**
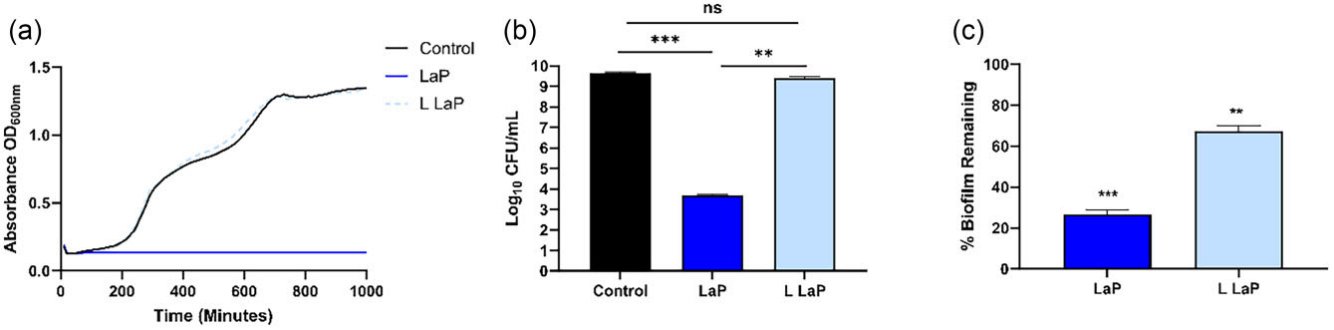
*Lactobacillus acidophilus* postbiotic (LaP) was bactericidal against planktonic *Ps. aeruginosa* and removes established biofilm *in vitro*. (A) *Pseudomonas aeruginosa* cultured with LaP derived from 1 × 10^8^ CFU ml^−1^ completely inhibited growth over 16 h, while LaP derived from 1 × 10^6^ CFU ml^−1^ (L LaP) had no effect (*n* = 4 independent experiments). (B) At the end of planktonic growth studies, *Ps. aeruginosa* cultures were spread onto TSA and colonies counted. LaP showed >5-log_10_ reduction in *Ps. aeruginosa* bacteria, while L LaP did not have a significant effect. (C) *Pseudomonas aeruginosa* biofilm was grown for 20 h before LaP and L LaP was added for 6 h. LaP and L LaP removed established biofilm by 74% and 26%, respectively (*n* = 4 independent experiments). Data are mean ± SEM % change from control. ***P < .001, **P < .01.

*Pseudomonas aeruginosa* or 20 h *Ps. aeruginosa* biofilm were challenged with low concentration LaP (L LaP; derived from 10^6^ CFU ml^−1^) or LaP (derived from 10^8^ CFU ml^−1^) and evaluated for the ability to reduce growth, kill planktonic bacteria, and remove established biofilm. LaP completely inhibited *Ps. aeruginosa* planktonic growth while L LaP had no affect on growth measured by absorbance compared to media only control (Fig. 1A). We then measured viable *Ps. aeruginosa* CFUs and found that LaP caused a 5.98-log_10_ reduction in CFU counts compared to media only control (Fig. 1B). Bactericidal activity (>99.9% inhibition) is defined as a >3-log_10_ reduction (Mascio et al. 2007, Keepers et al. 2014, Wookey et al. 2004). L LaP treatment resulted in only a 0.22-log_10_ reduction in *Ps. aeruginosa* (Fig. 1B). *Post hoc* analysis (Dunnett’s test) revealed a significant difference between LaP and media control (*P* < .0001) but not between L LaP and the media control (*P* = .331). As such, our data suggests that LaP is bactericidal against *Ps. aeruginosa*, and while L LaP may inhibit *Ps. aeruginosa* growth, it is not bactericidal. Moreover, LaP removed established *Ps. aeruginosa* biofilm (*P* = .0002), and L LaP was less effective (*P* = .005) (Fig. 1C). Therefore, only LaP (derived from 10^8^ CFU ml^−1^) was tested in subsequent studies to investigate properties of LaP *in vivo*, and for biochemical analyses.

In these initial studies, we determined if *Lact. acidophilus* LaP prepared in M63 minimal media versus LB media had different activity against *Ps. aeruginosa* biofilm activity, as both these media are commonly used in the growth of *Ps. aeruginosa*. Here, we demonstrate that LaP subcultured for 6 h in LB media instead of M63 minimal media did not have similar bactericidal or antibiofilm effects (Fig. S1A–C) suggesting that LaP prepared in M63 has specific antibacterial, immunostimulatory, and antibiofilm activity which is not found in LaP prepared in LB media.

### LaP treatment reduced pathogen burden in 24 h and 5 day wound infection

**Figure 2.**
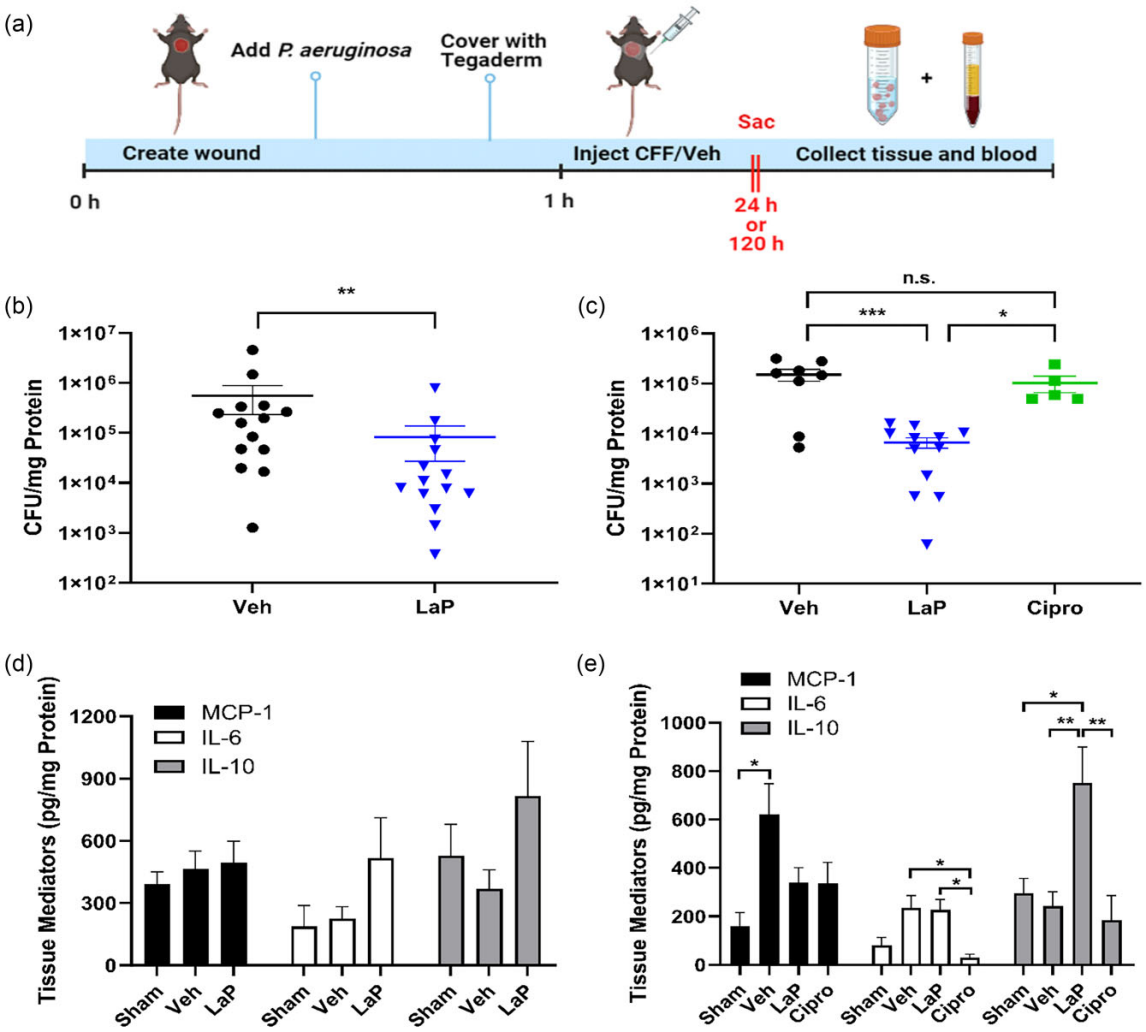
LaP reduces *Ps. aeruginosa* burden and modulates inflammation *in vivo*. (A) Schematic representation of mouse wound infection model. Full thickness excisional wounds were made on the backs of C57BL/6 mice. Wounds were infected with *Ps. aeruginosa* (10^7^ CFU) and secured with Tegaderm. 1 h later, M63 vehicle or LaP was injected into the wound and Ciprofloxacin (Cipro) was given s.c. (B) After 24 h, LaP significantly decreased *Ps. aeruginosa* burden compared to Veh (M63) control (*n* = 14 in each group). (C) LaP reduced *Ps. aeruginosa* bacterial load in 5 day wound infection (*n* = 8 Veh, *n* = 12 LaP, and *n* = 5 Cipro). (D) LaP treatment did not affect local tissue MCP-1, IL-6, or IL-10 levels following 24 h wound infection (*n* = 5 sham, *n* = 7 veh, and *n* = 8 LaP). (E) After 5 d, tissue MCP-1 and IL-6 levels were elevated in infected vehicle mice, but LaP treatment had no significant impact on either proinflammatory mediator production. On the other hand, IL-10 levels were increased by LaP treatment in the infected wound tissue (*n* = 5 sham, *n* = 6 veh, *n* = 9 LaP, and *n* = 5 Cipro). Data are mean ± SEM. ***P < .001, **P < .01, and *P < .05.

In wound tissue samples from sham, there was no measurable bacteria load (results not shown). LaP (derived from 10^8^ CFU ml^−1^) treatment significantly reduced bacteria load by 6.7 fold in 24 h wound infection (Veh control: 5.5 × 10^5^ versus LaP: 8.2 × 10^4^ CFU mg^−1^ protein) and by 48-fold in 5 day wound infection (Veh control: 3.1 × 10^5^ versus LaP: 6.4 × 10^3^ CFU mg^−1^ protein) compared to vehicle control (Fig. 2B and C, respectively). Unlike the single dose of LaP, the single dose of ciprofloxacin did not reduce wound bacteria load after 5 days (3 × 10^5^ CFU mg^−1^ protein; Fig. 2C).

### LaP did not alter wound tissue proinflammatory mediator levels but increased IL-10 secretion

We examined the effects of LaP on wound tissue levels of IL-6, a proinflammatory cytokine, MCP-1, a proinflammatory chemokine responsible for the migration of monocytes and macrophages, and IL-10, an antiinflammatory cytokine that modulates hyperinflammation (Short et al. 2022). After 24 h wound infection, there were no significant difference in tissue MCP-1, IL-6, and IL-10 levels between sham controls, infected vehicle, or LaP-treated mice, although there was a tendency for tissue cytokine levels to be higher in LaP-treated animals (Fig. 2D). After 5 days of wound infection, there was a significant increase in MCP-1 levels in infected vehicle controls compared to sham controls (Fig. 2E). However, there was no significant difference in MCP-1 in LaP-treated tissue compared to sham controls or infected vehicle controls (Fig. 2E). At 5 days, there was no significant difference between sham controls, vehicle-, and LaP-treated groups (Fig. 2E). Overall, LaP treatment had little impact on MCP-1 or IL-6 levels in 24 h or 5 day infected wound tissue. On the other hand, LaP-treated mice showed an ~3-fold increase in tissue IL-10 production (751.6 ± 147.8 pg mg^−1^ protein) following 5 day wound infection compared to vehicle controls (243 ± 59 pg mg^−1^ protein) and sham (295 ± 61.7 pg mg^−1^ protein) (Fig. 2E). Compared to vehicle control, ciprofloxacin did not alter tissue MCP-1 or IL-10 after 5 days (Fig. 2C). Interestingly, ciprofloxacin administration reduced tissue IL-6 levels 5 days after wound infection.

### LaP-modulated systemic inflammatory response in *Ps. aeruginosa* wound infection

**Figure 3.**
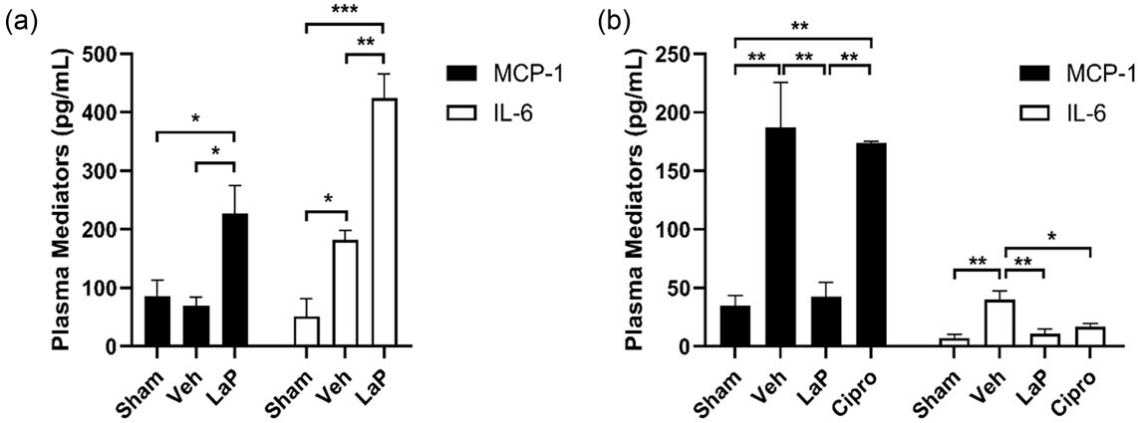
LaP increased plasma inflammatory mediator levels after 24 h but resolved systemic inflammation, 5 days after *Ps. aeruginosa* wound infection. (A) After 24 h, LaP treatment raised plasma MCP-1 and IL-6 levels compared to sham and infected vehicle controls (*n* = 5 animals for sham, *n* = 4 veh, and *n* = 3 LaP). (B) Following 5 day wound infection, infected mice treated with vehicle had increased MCP-1 and IL-6 levels compared to sham while LaP treatment decreased inflammation compared to infected vehicle (*n* = 3 for sham, *n* = 6 for veh, *n* = 5 for LaP, and *n* = 5 for Cipro). Data are mean ± SEM. ***P < .001, **P < .01, and *P < .05.

We measured the effect of LaP on plasma cytokine levels after 24 h and 5 days to investigate if LaP affected systemic inflammatory response to *Ps. aeruginosa* wound infection. After 24 h, plasma MCP-1 levels of infected vehicle were similar to that of sham controls but LaP treatment significantly stimulated production of MCP-1 (Fig. 3A). Similarly, 24 h after infection, plasma IL-6 levels of infected vehicle were higher than sham controls (Fig. 3A). Plasma from LaP-treated animals had greater IL-6 levels than both sham- and vehicle-treated mice. At 5 days postinfection, however, plasma MCP-1 levels in infected vehicle (187.2 ± 38 pg ml^−1^) were increased by >3-fold compared to sham controls (35 ± 8.6 pg ml^−1^) (Fig. 3B). LaP treatment completely abolished this increase and plasma MCP-1 levels (42.5 ± 17.4 pg ml^−1^) were similar to sham controls. After 5 days of wound infection, plasma IL-6 of infected control (40 ± 7.1 pg ml^−1^) was >4-fold greater than that of sham controls (7.2 ± 2.8 pg ml^−1^); Fig. 3B). LaP treatment reduced plasma IL-6 levels (10.9 ± 3.8 pg ml^−1^) to that of sham controls. These findings suggest that the LaP may first stimulate and then resolve inflammation *in vivo*. Compared to vehicle controls, ciprofloxacin treatment did not alter plasma MCP-1 levels but reduced plasma IL-6 levels to that of sham controls.

### LaP-stimulated adherence of THP-1 monocytes

**Figure 4.**
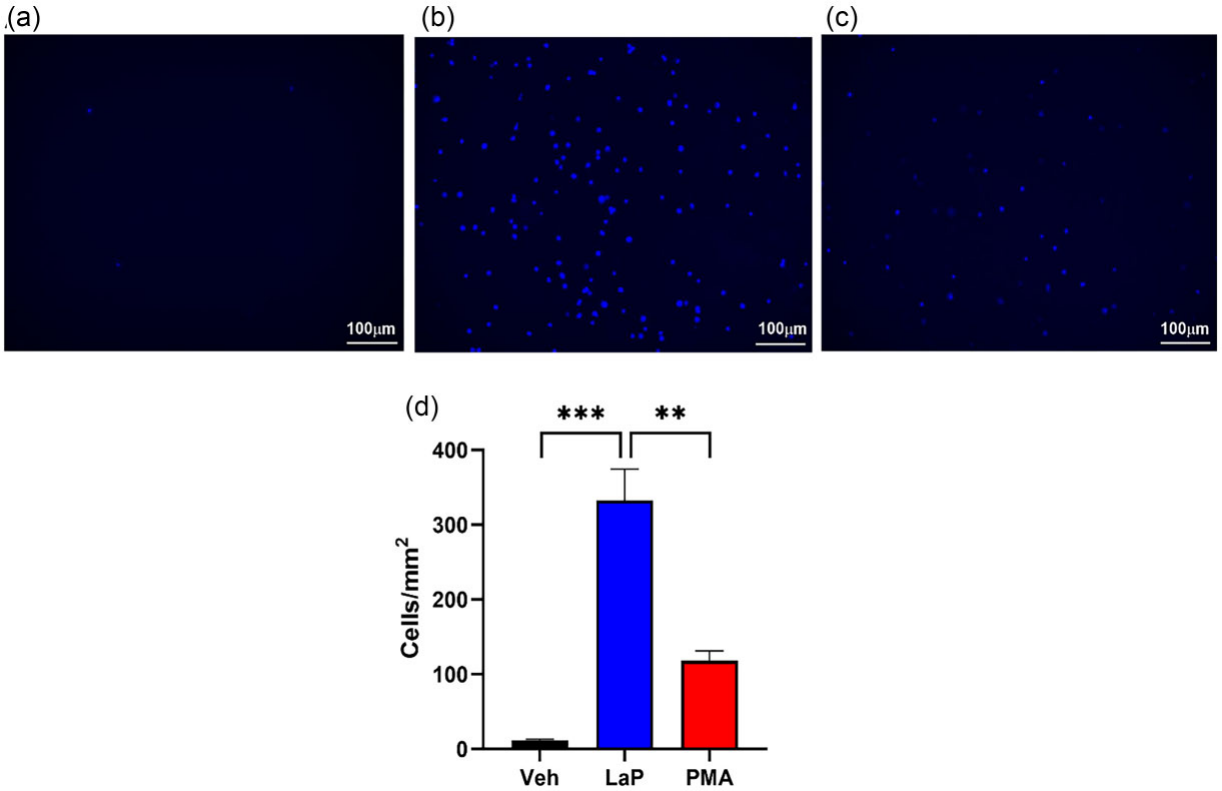
*Lactobacillus acidophilus* LaP increased THP-1 monocyte adherence. THP-1 monocytes were seeded in laminin-coated well plates. Either vehicle (M63; 5% v/v), LaP (5% v/v), or PMA (100 ng ml^−1^) was added for 1 h. At the end of incubation period, wells were washed and cells were stained with Cell-Brite blue and imaged for quantification by fluorescence microscopy and ImageJ analysis. (A) THP-1 monocytes treated with M63 vehicle. (B) THP-1 monocytes adhered to the well after LaP treatment. (C) PMA control also promoted monocyte adherence. (D) ImageJ quantification of monocyte adherence. LaP treatment significantly increased monocyte adherence compared to vehicle and to PMA controls. ***P < .001 and **P < .01; *n* = 3 independent experiments.

As monocyte migration and adherence are early steps to differentiation into macrophages, which are essential in tissue repair, we examined the possibility that LaP could activate monocytes to increase adherence. In these experiments, we incubated LaP with THP-1 monocytes for 1 h and measured adherence to laminin-coated plates. We show that monocytes do not adhere to the plate wells after only 1 h incubation with the vehicle control (Fig. 4A), but there was an ~300-fold increase in monocyte adherence after LaP treatment (Fig. 4B). Interestingly, this increase was significantly greater than the positive control PMA treatment (Fig. 4C and D). These data demonstrate the stimulatory activity of the LaP to increase monocyte adherence.

### SPE fraction 5 possesses antibiofilm activity

**Figure 5.**
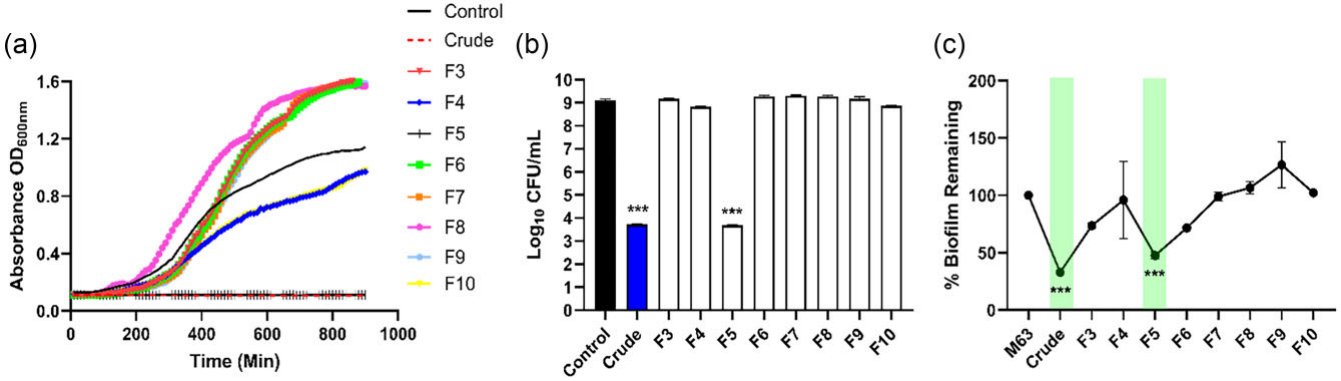
Planktonic growth and biofilm antagonistic performance of LaP SPE fractions. (A) *Pseudomonas aeruginosa* planktonic growth when incubated with LaP fractions for 16 h. Fraction 5 (F5) completely inhibited *Ps. aeruginosa* growth similar to crude LaP (dashed line). (B) Associated *Ps. aeruginosa* CFUs following 16 h growth curve study. Fraction 5 was the only fraction that had bactericidal activity (>5 log reduction in bacteria growth). (C) *Pseudomonas aeruginosa* biofilm remaining following LaP fractions incubation for 6 h. Shaded bars indicate crude LaP and active fraction (F5). *** *P* < .001, *n* = 3 independent experiments.

Following fractionation, lyophilization, and resuspension, the fractionated LaP was tested for antibacterial and antibiofilm activity. Fraction 5 completely inhibited *Ps. aeruginosa* planktonic growth (Fig. 5A) and was the only fraction that was completely bactericidal (Fig. 5B). It should be pointed out that some fractions (6, 7, 8, and 9) actually increased the number of CFU. Similarly, fraction 5 removed a significant amount of *Ps. aeruginosa* established biofilm (*P* < .001) (Fig. 5C). This activity was comparable to crude LaP (Fig. 5C), suggesting that the bioactive molecule is in fraction 5. Subsequent studies purified fraction 5 to elucidate the antibiofilm metabolite.

### HPLC fraction 24 removes established *Ps. aeruginosa* biofilm

**Figure 6.**
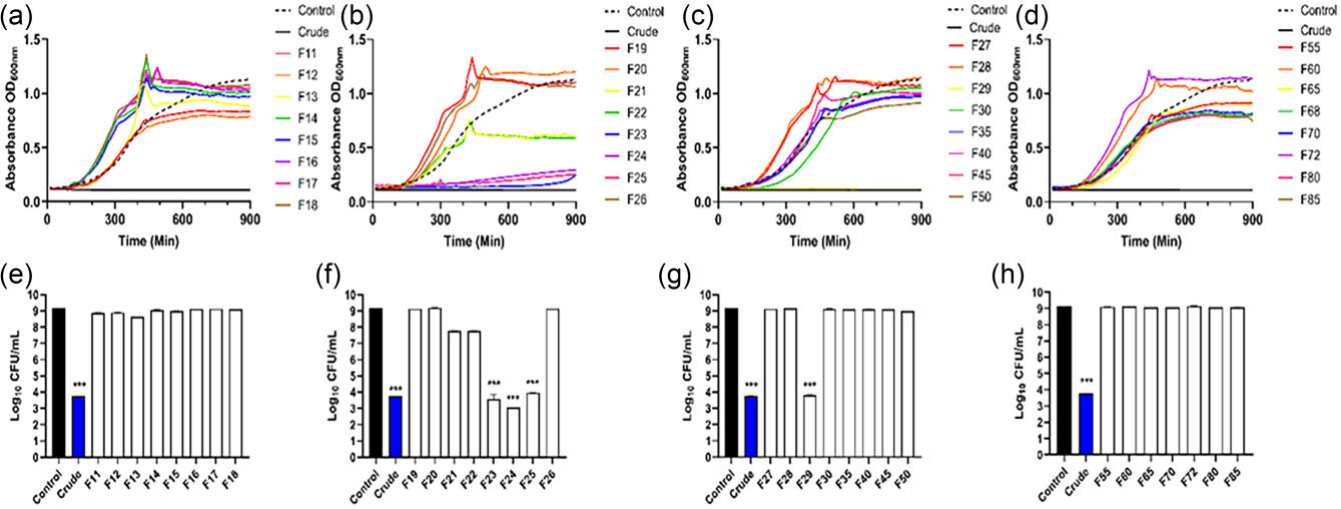
*Pseudomonas aeruginosa* planktonic growth with HPLC fractions of SPE fraction 5. Since fraction 5 collected from SPE significantly removed 20 h *Ps. aeruginosa* biofilm, fraction 5 was further fractionated to elucidate the potential antibacterial molecule using HPLC. (A–D) Planktonic *Ps. aeruginosa* growth with HPLC fractions for 16 h. Fractions 23, 24, 25, and 29 demonstrated the most reduction (>5 log reduction) in *Ps. aeruginosa* growth. (E–H) Associated CFUs measured after16 h growth curve studies. Data are mean ± SEM. *n* = 3 independent experiments.

**Figure 7.**
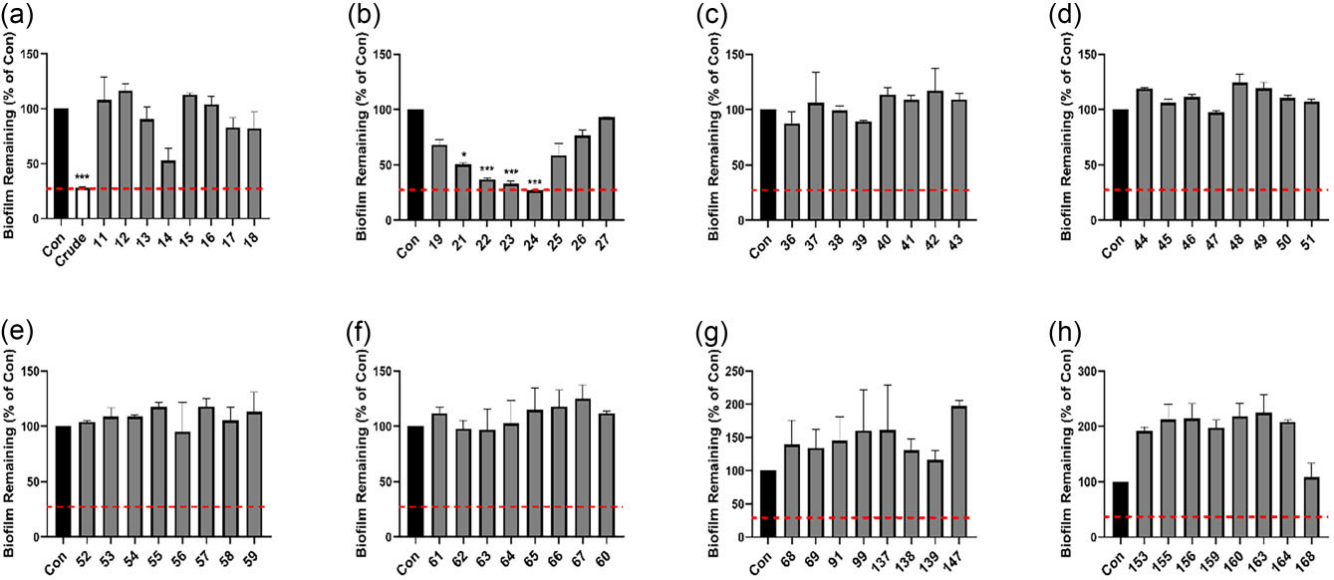
Antibiofilm activity of HPLC fractions. (A–H) *Pseudomonas aeruginosa* 20 h biofilm was subjected to 6 h treatment with HPLC fractions of Sep-Pak fraction 5 compared to crude LaP (indicated by horizontal dashed lined). HPLC fraction 24 showed similar biofilm removal activity to crude LaP. Several fractions increased the amount of biofilm formed compared to controls (>100%). Data are mean ± SEM. *P < .05, ***P < 0.001, and *n* = 4 independent experiments.

The SPE fraction 5 showed antibacterial and antibiofilm activity similar to crude LaP and was subjected to further fractionation using semipreparative HPLC over a 95/5 to 70/30 water/ACN containing 0.1% v/v formic acid gradient. Here, 168 fractions were collected, lyophilized, and resuspended in water and diluted in M63 minimal medium. Several fractions displayed *Ps. aeruginosa* planktonic growth inhibition (Fig. 6A–H) and were able to destroy 20 h established biofilm (Fig. 6A–H). Fractions 23, 24, and 25 possessed activity comparable to crude LaP (Fig 7). These fractions did not have peaks with long retention times indicating that these fractions had polar molecules This data is consistent with our previous findings that *Lact. acidophilus* secretes an antibiofilm molecule (Wilson et al. 2021). As such, we selected these three fractions (23, 24, and 25) for additional analysis using LCMS.

### Characterization of antibiofilm molecule found in HPLC fraction 24

In an effort to shed light on the bioactive metabolites within the LaP, we developed a fractionation and activity screening approach to investigate the metabolite profile of LaP fractions 23, 24, and 25 using LCMS. Other fractions collected from HPLC were also analyzed using LCMS to compare against active fractions and validate our approach. Since fractions 23, 24, and 25 possessed similar antibiofilm activity and chromatograms (data not shown), with fraction 24 demonstrating the highest biofilm removal ability, we reasoned that we could compare peak intensities across these three fractions and identify a peak with higher intensity in fraction 24. Interestingly, using this approach, only one hit with a higher intensity in fraction 24 compared to 23 and 25 was identified with a retention time of 0.43 min and a m/z of 295.1369 (data not shown).

We screened the MiMeDB database for metabolites with similar m/z to elucidate potential molecule classes and identities. Interestingly, the top 10 hits were hydroxy fatty acids, branched fatty acids and carboxylic acids, pteridines and derivatives, amino acid derivatives, phytoalexins, and terpene lactones (Table 1). These results suggest that fatty acids produced by *Lact. acidophilus* may be the bioactive molecules responsible for the antibacterial activity of the LaP.

**Table 1.** MiMeDB results for metabolites with similar m/z.

Name	Chemical formula	m/z	Monoisotopic mass (Da)	Class
2,3-Dihydroxy-3-methylpentanoate	C_6_H_11_O_4_	295.1387	147.0657	Hydroxy fatty acids
(R)-mevalonate	C_6_H_11_O_4_	295.1387	147.0657	Hydroxy fatty acids
Tetrahydropteridine	C_6_H_8_N_4_	295.139	136.0749	Pteridines and derivatives
*N*-methyl-l-dopa	C_10_H_13_NO_4_	295.1396	211.0845	Tyrosine and derivatives
*N*-methyl-d-dopa	C_10_H_13_NO_4_	295.1396	211.0845	Tyrosine and derivatives
3-Indoleacetonitrile	C_10_H_8_N_2_	295.1343	156.0687	Phytoalexins
Hericenone A	C_19_H_22_O_5_	295.1340	330.1467	Terpene lactones
Gibberellin A7	C_19_H_22_O_5_	295.1340	330.1467	C19-gibberellin 6-carboxylic acids
(R)-Pantoate	C_6_H_11_O_4_	295.1398	147.0663	Branched fatty acids
PGP(18:1(9Z)/14:0(3-OH))	C_38_H_74_O_14_P_2_	295.1410	816.4554	Phosphatidylglycerophosphates

The whole genome of *Lact. acidophilus* ATCC 4356 has been sequenced and determined to be 1 956 699 bp in length and contain 1977 coding sequences (Palomino et al. 2015, Dean et al. 2017). antiSMASH, a server designed to mine bacterial, fungal, and plant genomes for biosynthetic gene clusters (BGCs) that encode secondary metabolites can be used to compare across related species. Using antiSMASH, 12 distinct secondary metabolite genomic regions encoding for “ribosomally encoded and posttranslational modified peptides” (RiPPs), fatty acids, and saccharides were identified. Among these, 10 BGCs, including regulatory, transport, and core biosynthetic genes, were identified to produce saccharides. On the other hand, only two BGCs were observed for RiPP-like and fatty acid synthesis (results not shown). In parallel, MIBiG comparison (protocluster to region) associated 10 bacteriocins with the query sequence (Table 2). However, the associated bacteriocins had low similarity scores (<40%) (Table 2).

**Table 2.** List and size of associated bacteriocins and similarity to query sequence using MIBiG comparison.

Bacteriocin	Organism	Similarity score	Size (kDa)
Ubericin K	*Streptococcus uberis*	0.39	6.3
Lactococcin MN	*Lactococcus cremoris*	0.33	<10
Coagulin	*Bacillus coagulans*	0.32	8.6
Gassericin T	*Lactobacillus gasseri*	0.24	5.4
Gassericin E	*Lactobacillus gasseri*	0.24	5.0
Carnobacteriocin XY	*Carnobacterium maltaromaticum*	0.22	3.6
Lactococcin G	*Lactococcus lactis*	0.20	4.1
Salivaricin CRL1328 α	*Lactobacillus salivarius*	0.19	4.3
Sublancin 168	*Bacillus subtilis* subsp. *subtilis*	0.16	3.9

## Discussion

The search for antibiotic alternatives in combating AMR has increased within the past decade (Kumar et al. 2021). As a result, probiotics, prebiotics, and postbiotics have gained attention as prophylactic supplements, topical treatments, and oral or injectable therapies for a wide array of diseases and infections (Silva et al. 2020, Habteweld and Asfaw 2023, Rabetafika et al. 2023). Nonetheless, the mechanism of action, safety, and efficacy for pathogen control by probiotics is not completely established. Previously, we have shown that *Lact. acidophilus* postbiotic was bactericidal for *Ps. aeruginosa*, inhibited biofilm formation, and abolished 20 and 48 h established biofilm (Wilson et al. 2021). In addition, our lab (Wilson et al. 2021) and others (Rocha-Ramirez et al. 2017, De Marco et al. 2018) have demonstrated *in vitro* immunomodulatory properties of probiotic whole cell suspensions and cell-free supernatants. As such, the goal of this study was to examine the antibacterial and immunomodulatory effects in a mouse wound infection model and elucidate the bioactive metabolite(s) that possess such activities. To study this, we applied topical LaP, 1 h following *Ps. aeruginosa* wound infection and measured bacterial clearance, local tissue inflammatory mediator secretion, and systemic inflammation. Additionally, we used biochemical separation techniques and analyses to elucidate the identification of the antibacterial and antibiofilm metabolite. We found that LaP treatment reduced pathogen load in a mouse model of wound infection. LaP administration altered tissue and systemic inflammation. LaP also upregulated monocyte adherence showing that it may play a role in wound healing. We found that *Lact. acidophilus* secretes a low molecular weight molecule (m/z 295.1369) that is bactericidal against *Ps. aeruginosa* and removes 20 h established biofilm. For this reason, *Lact. acidophilus* postbiotic may be a promising antibiotic alternative that has bactericidal activity against *Ps. aeruginosa*, can destroy mature biofilm and modulate the inflammatory response *in vivo*.

In almost all reported *in vivo* studies, probiotics or postbiotics were administered prophylactically, before or at the time of infection, and therefore may not be therapeutically relevant (Chen et al. 2009, Khailova et al. 2013, Machairas et al. 2015, Argenta et al. 2016, Wang et al. 2020). Additionally, all studies focused almost completely on the antibacterial and survival effects of the probiotic or postbiotic with little attention to systemic inflammatory effects. Here, we showed that LaP treatment decreased *Ps. aeruginosa* bacterial burden by ~8-fold in 24 h wound infection and reduced bacterial load by ~10-fold, 5 days after wound infection. This result suggests that the antibacterial mechanisms of LaP started early and continued over 5 days.

Interestingly, ciprofloxacin given once at 1 h after the wound infection did not significantly reduce tissue bacterial burden after 5 days. The results are probably due to the fact that ciprofloxacin efficacy is dependent on multiple doses (Laulund et al. 2022) In the studies of Laulund et al. (2022), ciprofloxacin was administered at ~1 mg per day per mouse s.c. against *Ps. aeruginosa* biofilm for 8 days. This regimen reduced bacteria load by 2 orders of magnitude. We used a single dose of ~6.25 mg per mouse and sacrificed the mice 5 days later, so the total dose of ciprofloxacin in our study (over 5 days) was equivalent to that of Laulund et al. (2022). Thus, it is possible that the inability for ciprofloxacin to kill significant bacteria numbers may be due to the need for multiple doses. Another possible cause is due to ciprofloxacin resistance. It has been reported that the *Ps. aeruginosa* strain ATCC #27853 used here, can acquire ciprofloxacin resistance quite rapidly after a single dose (Sihotang et al. 2022) Our results therefore suggest that a single dose of LaP applied at the same time as ciprofloxacin was given, was more efficacious in reducing *Ps. aeruginosa* wound infection. This provides proof of principle that topical LaP is an efficacious antibacterial *in vivo*.

In the early stages of wound infection, inflammation is necessary to clear pathogens and promote wound healing. However, overzealous inflammation can result in delayed wound healing and potential tissue damage. This highlights the need for inflammation resolution. In our studies, wound tissue MCP-1, IL-6, and IL-10 levels were not significantly altered after 24 h of infection and LaP treatment tended to increase tissue IL-6 levels. At 5 days, MCP-1 tissue levels were upregulated in vehicle controls, while wound tissue from LaP animals were similar to sham controls, although not significant, infected vehicle controls had a tendency to have higher tissue IL-6 and LaP tended to reduce tissue levels of this inflammatory cytokine. Furthermore, IL-10 (antiinflammatory cytokine) levels were elevated in LaP-treated mice after 5 days. Our results suggest that in infected vehicle controls, there was increased tissue inflammation consistent with the established hyperinflammation infection paradigm (Rello et al. 2017). Our results also indicate that LaP may first have immunostimulatory properties to clear infection and later resolves inflammation. It should be noted that IL-10 is a key cytokine responsible for epithelial and mucosal repair (Quiros et al. 2017, Saraiva et al. 2020) providing evidence that the LaP may promote tissue repair mechanisms.

In our studies, LaP treatment stimulated MCP-1 and IL-6 production in plasma after 24 h wound infection. This data confirms a previous report demonstrating that *L. plantarum* treatment increased serum IL-6 levels after 24 h *S. aureus* wound infection in rats (Ong et al. 2020). These results are completely consistent with our previous *in vitro* studies, which showed that LaP-simulated TNF-α release in LPS-stimulated THP-1 monocytes (Wilson et al. 2021). In these *in vitro* studies, we showed that LaP increased activity of the transcription factor, NF–κB activity in THP-1 monocytes, strongly suggesting that the mechanism for the increased TNF-α release was through the activation of NF–κB. This result provides support that the mechanism by which *Lact. acidophilus* LaP stimulates systemic inflammatory cytokine and chemokine production is by upregulating NF–κB activity. After 5 days, plasma MCP-1 and IL-6 levels were significantly increased in infected vehicle mice compared to sham controls. At this time point, LaP treatment attenuated plasma MCP-1 and IL-6 levels compared to infected vehicle mice and were similar to sham controls. Once again, this result is consistent with our *in vitro* study, which showed that lower concentration LaP reduced NF–κB activity and TNF-α production in LPS-stimulated THP-1-derived macrophages (Wilson et al. 2021). We postulate that LaP modulates the host response by initially stimulating the immune response to clear infection, but over time, in the highly stimulated cells, the now diluted and lowered concentration of LaP resolves and reduces the inflammatory response. The effect of LaP treatment on upstream regulators of NF–κB activation, such as toll-like receptor (TLR)-4 and TLR2 should be investigated to determine the direct immunomodulatory effects on host monocytes and macrophages.

Interestingly, ciprofloxacin significantly decreased tissue and plasma IL-6 levels but did not alter MCP-1(tissue and plasma) or tissue IL-10 levels. MCP-1 is a chemotactic cytokine (Singh et al. 2021), and therefore increased MCP-1 levels would be associated with the high bacteria load observed with ciprofloxacin administration. IL-6, however, is a proinflammatory cytokine produced after infection or injury. Ciprofloxacin mediated reduction in IL-6 levels may be due to direct inhibition of cellular production of cytokines such as IL-6 (Silva Lagos et al. 2022). In addition, there are reports that MCP-1 can be elevated without significant increase in other cytokines such as Il-6 in various situations such as SARS CoV-2 infection of septic patients (Eichhorn et al. 2023) and chronic fatigue syndrome (Groven et al. 2020). These reports coupled with our results, suggest that ciprofloxacin was able to reduce IL-6 levels without affecting cellular ability to produce MCP-1.

Monocyte migration and subsequent differentiation into macrophages is a critical process in the inflammatory stage of the wound healing process (Raziyeva et al. 2021). In our studies, we show that LaP increased monocyte adherence *in vitro*. As monocyte adherence is a well-established early step in monocyte differentiation into macrophages (Yang et al. 2014, DiPietro et al. 2021), it is plausible that a mechanism by which LaP may increase tissue repair is by activating monocytes. As a result, this activation may lead to IL-10 production and enhanced wound healing. A limitation to our study is we were unable to examine the effects of LaP on tissue and/or cellular changes such as monocyte, fibroblast, keratinocyte, or collagen deposition within the wound through histology. This was because we were using tissue samples for bacteria and cytokine measurements.

There are several potential bioactive compounds and mechanisms, which may be implicated in the antibacterial properties of probiotics. Importantly, *Lact. acidophilus* utilizes quorum sensing, specifically the LuxS/AI-2 system to promote survival in acidic conditions, including the stomach, as well as producing antimicrobial compounds (Meng et al. 2022). There are currently no studies demonstrating that laboratory culture conditions directly impacted *Lact. acidophilus* bacteriocin or acid production. However, we have evidence that minimal media conditions, such as M63 minimal media, induces *Lact. acidophilus* to secrete bioactive molecules. In the present study, LaP preparation was optimized to collect metabolites that had antibacterial properties against *Ps. aeruginosa*. In our studies, *Lact. acidophilus* was cultured in MRS broth for 48 h as recommended (Besser et al. 2019, Han et al. 2019, Maghsood et al. 2020), and the cells were washed, resuspended, before being subcultured for 6 h in M63 minimal medium (Wilson et al. 2021). Interestingly, most investigators using lactobacilli or lactobacilli cell-free supernatants to study its antibacterial activity made their preparations with MRS media (Kim et al. 2021, Raheem et al. 2021). This is a specific media, which allows *Lactobacillus* spp. to grow abundantly (Dave and Shah 1996, Oleksy-Sobczak and Klewicka 2020) and possibly out compete other microorganisms. We previously reported that MRS media alone can reduce *Ps. aeruginosa* biofilm formation (Wilson et al. 2021). Interestingly, subculturing *Lact. acidophilus* in LB broth did not result in significant *Ps. aeruginosa* killing or biofilm removal while subculturing in M63 minimal media showed bactericidal activity, biofilm formation inhibition, and eradication of mature biofilm (Fig. 1). Interestingly, there are reports documenting that culture conditions can impact the growth and metabolic activity of *Lact. acidophilus* (Corcoran et al. 2005). In our previous study, we showed that HCl, which had been acidified to a similar pH as the LaP (pH = 4) did not have significant antibiofilm or antibacterial effects (Wilson et al. 2021). Therefore, pH *per se* or alone cannot account for the activity of the LaP. However, it is possible that the pH of the LaP plays a role in its activity. The pH of the LB was ~6.8 suggesting that acidity of the media may be an important factor in its activity. It is thus plausible that subculturing *Lact. acidophilus* in M63 minimal medium may lead to increased bioactive metabolite production, or allow certain low pH bioactive metabolites to exist.

Bioinformatic analysis using antiSMASH identified a BGC encoding for fatty acids and screening the MIBeB database for molecules with similar m/z (Table 2) provided support that the antibiofilm metabolite may be a fatty acid. Conjugated fatty acids and hydroxy fatty acids are known to be modified by probiotic bacteria through fermentation and biohydrogenation (Manzo et al. 2015, Kuhl and Lindner 2016, Peng et al. 2018, Zhou et al. 2018, Lashkari et al. 2020). However, there is little evidence showing that probiotic bacteria make and secrete polyunsaturated fatty acids (PUFAs). Most organisms, including humans, cannot synthesize long chain PUFAs (Jovanovic et al. 2021, Shah et al. 2022). Intriguingly, some groups of bacteria produce PUFAs, such as docosahexaenoic acid (DHA), arachidonic acid, and eicosapentaenoic acid (EPA) through anaerobic *de novo* synthesis using polyketide synthases or PUFA synthases (Metz et al. 2001, Yoshida et al. 2016, Moi et al. 2018).

Peng et al. (2018) engineered *L. casei* to enhance CLA production and observed reduced *Salmonella enterica* serovar Typhimurium and enterohaemorrhagic *E. coli* biofilm formation. In addition, 5 week pretreatment with a PUFA mixture (DHA/EPA) prior to *Ps. aeruginosa* pulmonary infection in mice decreased mortality and *Ps. aeruginosa* bacterial load (Caron et al. 2015). LA, CLA, and other PUFAs may exert antibacterial properties by inhibiting pathogen DNA replication, efflux pumps, metabolism, bacterial cell wall biosynthesis, quorum sensing, or by disrupting the cell membrane (Sun et al. 2016, Le and Desbois 2017, Casillas-Vargas et al. 2021). Our studies did not directly measure CLA and other PUFA presence within LaP, however, bioinformatic analyses indicated that several hydroxy fatty acids have m/z similar to our antibiofilm metabolite. Moreover, in our experiments, *Lact. acidophilus* was subcultured and LaP collected in M63 minimal medium supplemented with 1 mM MgSO4, 0.2% glucose, and 0.5% casamino acids, and we postulate that these culture conditions alter *Lact. acidophilus* metabolism, and therefore may provide substrates for PUFA *de novo* synthesis.

Probiotic bacteria, including *Lact. acidophilus* are known to produce lactic acid, short-chain fatty acids such as butyrate and acetate, and AMPs, namely bacteriocins (Indira et al. 2019). Short-chain fatty acids are carboxylic acids with fewer than six carbon atoms that are produced by microbiota through the fermentation of dietary fibers and carbohydrates (Usta-Gorgun and Yilmaz-Ersan 2020). Recently, it has been reported that acetic acid caused over 19% *Ps. aeruginosa*-induced cell shrinkage and disruption of biofilm matrix (Tawre et al. 2021). In contrast, we have shown that acetic acid alone removed 20 h established *Ps. aeruginosa* biofilm, but not to the same extent as LaP (Wilson et al. 2021), suggesting that acetate produced by *Lact. acidophilus* is not solely responsible for antibacterial effects.

AMPs are small peptides that may be promising alternatives to antibiotics and have been reported to have activity against MDR bacteria, including *Ps. aeruginosa*, and some may also promote wound healing, angiogenesis, and modulate the immune response (Zhang et al. 2021). AMPs function as antimicrobials by targeting pathogen cell membranes while preserving host cells (Enoki et al. 2018, Zhang et al. 2021). Among the many documented AMPs, bacteriocins in particular are known to be produced by various probiotics, such as *Lactobacillus* spp. For example, *Lact. gasseri* LA327 has been reported to produce gassericin T and gassericin S, two bacteriocins that have antibacterial activity against *Bacillus cereus* and *Ps. fluorescens* (Arakawa et al. 2009, Kasuga et al. 2019). Importantly, bacteriocins tolerate a wide pH range, (2.0–11.0) (Kasuga et al. 2019, Darbandi et al. 2022) and therefore could withstand the acidic conditions of LaP (pH 4.0–5.0). In contrast, most bacteriocins do not tolerate proteolysis (Elayaraja et al. 2014, Kasuga et al. 2019, Darbandi et al. 2022), although some have been reported to be protease resistant (Singh et al. 2014, Ansari et al. 2018). Our previous data showed that LaP treated with Proteinase K did not significantly impact the biofilm removal ability (Wilson et al. 2021). Moreover, using antiSMASH in our bioinformatic analyses, we identified the top 10 similar BCGs encoding for bacteriocins, including ubericin K and gassericin T, gassericin S, and the similarity was low (<35% similarity). This low score means that only one-third of genes are matching with BGCs of known RiPPs. Regardless, antismash identifies RiPP BGCs. It may not be unexpected that RiPP BGCs show low similarity because they encode for many modifying enzymes that are different between BGCs. We think it is highly unlikely that the observed activity stems from a bacteriocin however, since we did not observe any obvious mass spectrometry signal for a larger (3–10 kDa) molecule and peptidic molecule. We used ESI with a very low cone voltage and temperature (soft ionization), causing very minimal fragmentation (if at all). A typical bacteriocin is in the range of 3–10 kDa. For these kinds of peptidic molecules, we should observe a typical mass-to-charge ratio profile in which the molecule is multiply charged (Maky et al. 2021). In addition, fragmentation by MS/MS would show peptide bond cleavage. We did not observe any of these characteristic hallmarks of bacteriocin mass spectrometry. Taken together, our findings with proteinase K coupled with our LCMS and bioinformatics work suggest that the biofilm removal metabolite within the LaP is unlikely to be a bacteriocin.

In summary, our studies suggest that LaP topically applied a full hour after bacterial infection reduced pathogen load and modulated the inflammatory response in a mouse wound infection model. The mechanism by which LaP may exert its beneficial effects is by a direct antibacterial action as well as through modulating monocyte/macrophage function. Coupled with our previous and current *in vitro* studies, the potential bioactive molecule is likely a low molecular weight, polar metabolite that has a m/z of 295.1369. However, further isolation and MS/MS studies are needed to purify the metabolite, elucidate the identity, and confirm its activity *in vitro* and *in vivo*. These findings provide support for the potential use of *Lact. acidophilus* postbiotic as an antibacterial alternative during wound infection.

## Supplementary Material

lxaf061_Supplemental_Files

## Data Availability

The data reported in this study are available from the corresponding author upon reasonable request.
