# Supplementary figures and images for: Lactobacillus acidophilus (strain Scav) postbiotic metabolites reduce infection and modulate inflammation in an in vivo model of Pseudomonas aeruginosa wound infection
Source: J Appl Microbiol. 2025 Mar 11;136(3):lxaf061. doi: 10.1093/jambio/lxaf061 (PMC11951090; doi:10.1093/jambio/lxaf061)

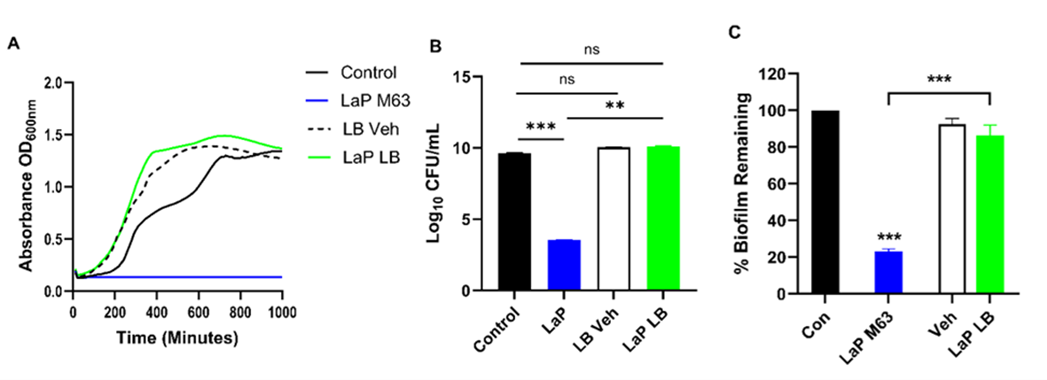

Supplement: lxaf061_Supplemental_Files [file lxaf061_supplemental_files.zip › Figure S1.tif]

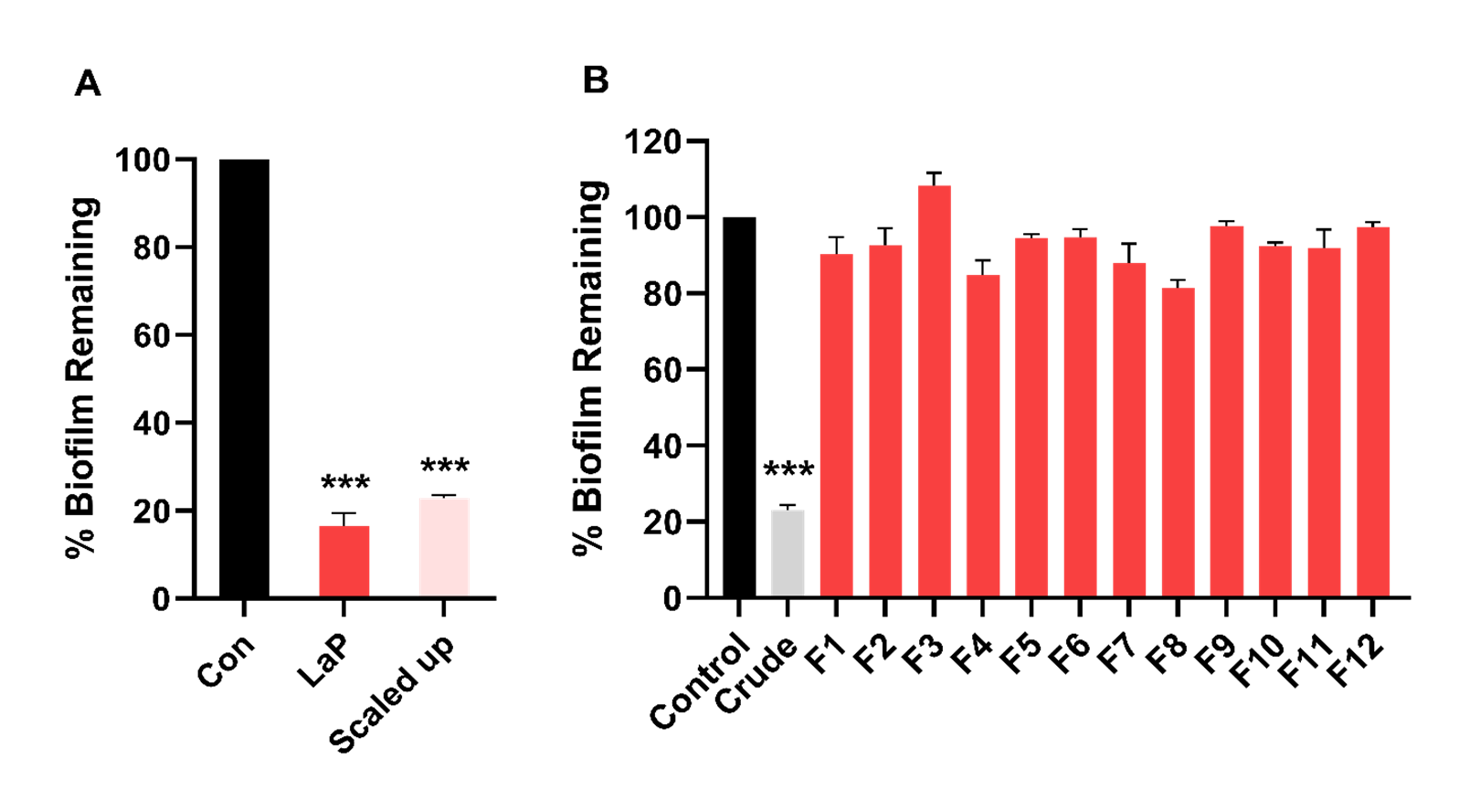

Supplement: lxaf061_Supplemental_Files [file lxaf061_supplemental_files.zip › Figure S2.tif]
